# Arrhythmogenic superior vena cava manifesting after a right‐sided pneumonectomy and mediastinal lymph node dissection

**DOI:** 10.1002/joa3.12804

**Published:** 2022-12-27

**Authors:** Kanae Hasegawa, Shinsuke Miyazaki, Akitoshi Okada, Tomokazu Ishida, Hiroshi Tada

**Affiliations:** ^1^ Department of Cardiovascular Medicine, Faculty of Medical Sciences University of Fukui Fukui Japan; ^2^ Division of Thoracic Surgery, Department of Surgery, Faculty of Medical Sciences University of Fukui Fukui Japan; ^3^ Department of Radiography, Faculty of Medical Sciences University of Fukui Fukui Japan

**Keywords:** atrial fibrillation, catheter ablation, lung cancer, mediastinal lymph node dissection, pneumonectomy

## Abstract

No case of AF ablation after right‐sided pneumonectomy has been reported, presumably because the pneumonectomy renders the ablation procedure more difficult than lobectomy because of the marked mediastinal displacement. In the case of catheter ablation of AF after right‐sided pneumonectomy, it is extremely important to insert a mapping catheter not only into the PV but also into the SVC to accurately diagnose the site of abnormal electrical activity.

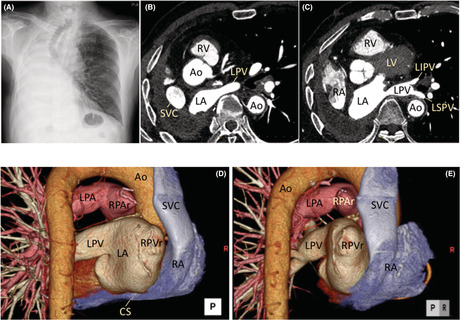

## 1 SPOTLIGHT

**FIGURE 1:**
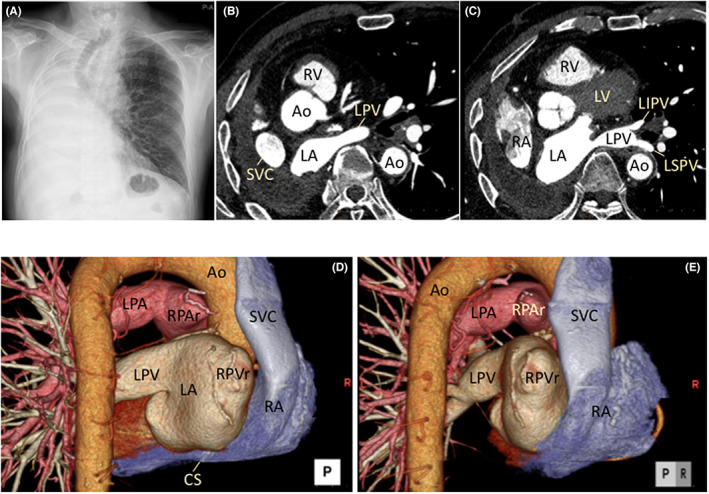
(A) Chest X‐ray showing the heart, bronchus, and esophagus, all displaced to the right after the lung cancer surgery. (B, C) Computed tomography scan revealing that the heart was deviated to the right. (D, E) Three‐dimensional reconstructed imaging on computed tomography showing the remnant of the right PV in contact with the SVC. The left PV had a common trunk. Ao, Aorta; CS, coronary sinus; LA, left atrium; LPA, left pulmonary artery; LI(S)PV, left inferior (superior) pulmonary vein; LPV, left pulmonary vein; RA, right atrium; RPAr, remnant of the right pulmonary artery; RPVr, remnant of the right pulmonary vein; SVC, superior vena cava.

A 77‐year‐old man suffered from palpitations for 1 month for the first time and underwent an ablation procedure for drug‐refractory paroxysmal atrial fibrillation (AF). Nine years prior, because of lung cancer, the patient underwent a complete right‐sided pneumonectomy with a cut vessel sheath to bare the right PV and artery, and a mediastinal lymph node dissection with a cut vessel sheath to bare the SVC and part of the pericardium. The physical examination was unremarkable. An echocardiogram revealed normal left ventricular systolic function and normal left atrial (LA) size (LA diameter = 35 mm). A chest X‐ray (Figure 1A) and three‐dimensional reconstruction imaging of the computed tomography (Figure 1B–E) showed a marked displacement of mediastinal structures to the right, a remnant of the right PV, and a left common PV.

**FIGURE 2:**
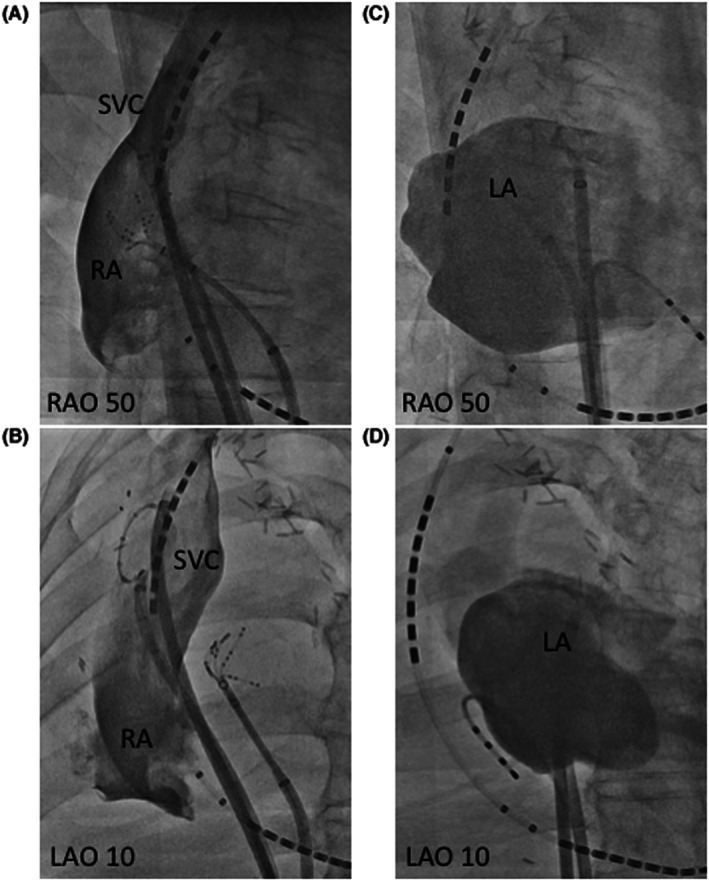
(A, B) Angiography of the RA and SVC. (C, D) Angiography of the LA and RPVr stump. LA, left atrium; LAO, left anterior oblique view; RA, right atrium; RAO, right anterior oblique view; RPVr, remnant of the right pulmonary vein; SVC, superior vena cava.

Catheter ablation was performed under moderate sedation obtained with dexmedetomidine. The triggers of AF were explored with multipolar mapping catheters during the repetitive appearance of spontaneous AF following a single transseptal puncture using a radiofrequency needle under the guidance of intracardiac echocardiography. Angiography of the SVC (Figure 2A,B) and LA (Figure 2C,D) was also performed to clarify the cardiac anatomy.

**FIGURE 3:**
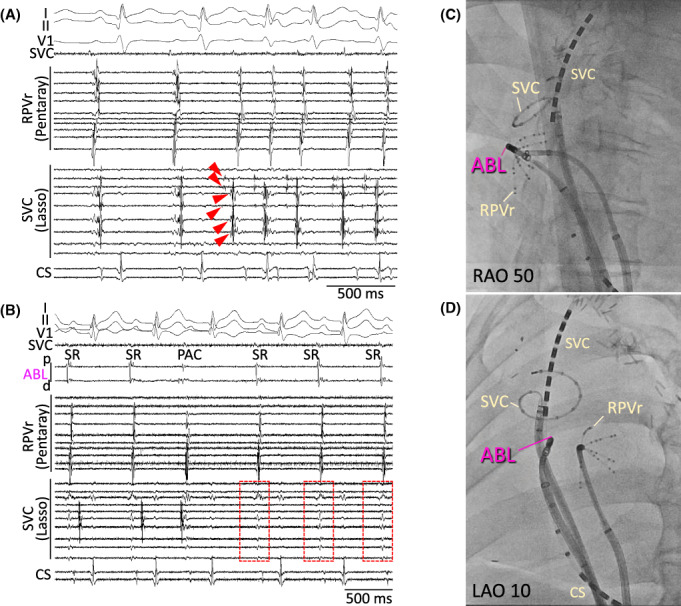
(A) A circular mapping catheter was placed in the SVC and a multipolar mapping catheter (Pentaray®, Biosense Webster) was positioned at a remnant of the right PV stump. AF initiation from the SVC (arrows) was identified. (B) The SVC potentials were abolished when the electrical SVC isolation was achieved. (C, D) the catheter position and successful ablation site on fluoroscopy. ABL, ablation catheter; AF, atrial fibrillation; CS, coronary sinus; LAO, left anterior oblique view; RA, right atrium; RAO, right anterior oblique view; RPVr, remnant of the right pulmonary vein; SVC, superior vena cava.

No PV potentials were identified in the left common PV, and no low voltage area was identified in the LA. Mapping with a circular mapping catheter (Lasso®, Biosense Webster, Irvine, CA) and a multipolar mapping catheter (Pentaray®, Biosense Webster) revealed that AF was reproducibly elicited from repetitive electrical activities within the SVC (Figure 3A). Single premature electrical activity also occurred within the remnant of the right PV stump, but AF was never elicited from this premature activity. An electrical SVC isolation was successfully performed with an irrigated‐tip ablation catheter (ThermoCool Smarttouch® Surroundflow Catheter, Biosense Webster) without any complications (Figure 3B–D). The isolation of the right PV stump was performed under the guidance of a three‐dimensional mapping system. After the SVC isolation, isolated dissociated electrical activity was observed within the SVC; AF no longer appeared spontaneously and was not induced during an isoproterenol infusion. The outpatient clinic visits were at 1 and 3 months post‐procedure and every 2–3 months thereafter, and subsequent visits consisted of a clinical interview and 3‐min ECGs at our cardiology clinic. As of the 26‐month follow‐up, the patient has remained free from any atrial arrhythmias without any antiarrhythmic drug use.

## 2 DISCUSSION

This is the first case in which an arrhythmogenic SVC has been successfully isolated after a complete right‐sided pneumonectomy and mediastinal lymph node dissection for lung cancer 9 years prior.

PVs are well‐known sources of AF, and the SVC is the most common non‐PV focus in patients with paroxysmal AF.^1^ Several reports have described the arrhythmogenicity of the remnant PV stump, that is, premature or repetitive electrical activities originating from this site can induce AF.^2^, ^3^, ^4^, ^5^ Forty‐two patients underwent lobectomy and 6 underwent a complete left‐sided pneumonectomy^2^, ^3^, ^4^; however, in these reports, there was no indication of lymph node dissections.^2^, ^3^, ^4^ The incidence of AF post‐surgery was higher after a pneumonectomy than after a lobectomy, and the risk factor of postoperative AF included mediastinal lymph node dissection.^5^ However, there have been few reports on AF ablation after a pneumonectomy. In particular, no case of AF ablation after right‐sided pneumonectomy has been reported, presumably because the pneumonectomy renders the ablation procedure, particularly related to the left atrium, more difficult than lobectomy because of the marked mediastinal displacement.

The exact mechanism of abnormal electrical activity of the SVC in this case is unknown. In fact, only after the cardiac surgery, did the pathophysiological analysis of the atrial tissue samples reveal that the surgical trauma induced an arrhythmogenic substrate during the perioperative period, and the atriotomy and/or inflammation induced an arrhythmogenic substrate during the acute postoperative period. However, any tissue with myocardial sleeves, such as the SVC were not examined and mentioned. Moreover, in this patient, AF was observed for the first time 9 years after a pneumonectomy, so there may have been additional chronic stimuli, such as traction from a marked displacement or the traction from the SVC on the surrounding tissues. As suggested by the mechanism of the abnormal electrical activity in the remnant PV stump, the surgical injury, ischemia, inflammation of the SVC, and marked displacement of the mediastinum, including of the heart and SVC, may have promoted arrhythmogenesis because of the fibrotic and anisotropic changes within the myocardial SVC sleeve. In addition to the complete right‐sided pneumonectomy, a mediastinal lymph node dissection with a cut vessel sheath to bare the SVC was performed, which may have caused more directly surgical damage to the SVC.

## 3 CONCLUSIONS

In this case, detailed and simultaneous mapping of the SVC and right PV remnant was useful in diagnosing abnormal electrical activity in the SVC, and electrical isolation of the SVC resolved the AF. In the case of catheter ablation of AF after right‐sided pneumonectomy, it is extremely important to insert a mapping catheter not only into the PV but also into the SVC to accurately diagnose the site of abnormal electrical activity.

## CONFLICT OF INTEREST

There are no conflicts of interest.

## DECLARATIONS

*Approval of the research protocol*: Research Ethics Committee of University of Fukui (No.20180040). *Informed consent*: The patient provided informed consent. *Registry and the registration no. of the study/trial*: N/A. *Animal studies*: N/A.
